# Assessing Energy Availability and Glucose Dynamics in Adolescent Cyclists: Implications for Nutritional Interventions During the Competitive Season

**DOI:** 10.3390/nu16223824

**Published:** 2024-11-07

**Authors:** Matteo Tarocchi, Alessio Pellegrino, Kristina Skroce, Andrea Zignoli, Letizia Clara Cavadini, Chiara Bodini, Giuditta Pagliai, Loira Toncelli, Laura Stefani, Simone Vanni, Maria Boddi, Alessandra Modesti, Pietro Amedeo Modesti

**Affiliations:** 1 Department of Experimental and Clinical Medicine, University of Florence, 50134 Florence, Italy; 2 Sports Medicine Unit, Careggi University Hospital, 50134 Florence, Italy; 3 Supersapiens Inc., Atlanta, GA 30318, USA; 4 Department of Experimental and Clinical Biomedical Sciences, University of Florence, 50134 Florence, Italy

**Keywords:** energy availability, nutrition, endurance, athletes, adolescent, cycling, glucose monitoring, supplementation, environment, temperature

## Abstract

Background: The risk of developing a state of low energy availability (LEA) (<30 kcals/kg free-fat mass) in endurance athletes is known and recommendations for nutrition are available. However, information on male adolescent cyclists and the influence of hot temperatures is limited. Objectives: The aim of this study was to investigate the impact on energy availability of two 4-day nutritional intervention strategies: (1) supplementary carbohydrate (CHO) intake during exercise and (2) designing and implementing individual nutritional interventions. Methods: Each intervention was preceded by a 4-day basal assessment. Eight competitive male junior road cyclists (aged 16–17 years) were investigated using a 4-day diet and activity records, alongside bioelectric impedance analysis. Their real-time power output, interstitial glucose, and temperature were recorded via sensors and a bike computer. Their energy intake (EI) was estimated from daily, self-reported food diaries. Results: Overall, 100% and 71% of the cyclists were in a state of LEA during the baseline assessment of the supplementary CHO and nutritional interventions, respectively. LEA prevalence, not modified by supplementary CHO intake alone (from 100% to 87%, ns), was markedly reduced by the individual nutritional intervention (from 71% to 14%, *p* < 0.05). When considering all the data as a whole, LEA was positively influenced by the training load (OR 1.06; 95% Cl 1.03 to 1.09) and free-fat mass (OR 1.46; 1.04 to 2.04) and was negatively affected by EI (OR 0.994; 0.991 to 0.997). A hot environment (air temperature) failed to influence the LEA or glucose dynamics. Conclusions: the nutritional intervention, but not the supplementary CHO intake, markedly reduced the prevalence of LEA in adolescents, who often fail to match their energy expenditure with their energy intake during the competitive season. Nutritional education is essential for adolescent endurance cycling teams.

## 1. Introduction

Endurance athletes require carbohydrate intake at regular intervals during exercise [1] to ensure adequate availability of energy for consumption by the muscles [2,3,4,5] and to limit fatigue [1]. The American College of Sports Medicine guidelines currently take into consideration the intensity and duration of the exercise as a guide to consuming supplementary carbohydrates (CHOs) during exercise and in the recovery phase, in order to fully replenish the glycogen stores [2,6,7]. However, cyclists are often fearful of causing an increase in their body weight that could limit the weight/power ratio and often adopt dietary strategies aimed at limiting their body weight [8]. Energy intake (EI) that does not compensate for the exercise energy expenditure (EEE) may, however, cause a reduction in energy availability (EA), namely the amount of food energy available to support physiological functions after subtracting the energy cost of the exercise in relation to the free-fat mass (FFM) [9]. Dietary interventions solely focused on weight control can, thus, upset this fragile balance, with the possibility that a cyclist in training may experience low energy availability (LEA). Athletes are at risk of adopting dietary habits influenced by myths, misinformation, and marketing that often prioritize the use of food supplements over proper nutrition [10]. This point is crucial for adolescent athletes [11]; also, because at this age, the foundations for a healthy lifestyle and adult health are created [12].

The hypothesis in the present study is that a lack of knowledge or a voluntary restriction of carbohydrate intake may contribute to LEA in adolescent athletes. Therefore, the aims of this study were: (1) to assess energy availability and glucose dynamics in adolescent elite cyclists and (2) to compare the effects of a supplementary food correction during exercise and a global nutritional intervention, in adolescent athletes in different phases of the competitive season.

## 2. Materials and Methods

### 2.1. Subjects Investigated

Eight highly trained male junior road cyclists, belonging to the same team and identified as Tier 3 athletes or above [13], were investigated. The exclusion criteria were non-eligibility for cycling competitions [14], type 1 or type 2 diabetes, and temporary conditions involving the suspension of sports activity. The procedures of the study were carefully explained to each adolescent and their parents, who provided written informed consent. The study was approved by the local Ethics Committee (Ref. CE 22843_oss) on 19 March 2024.

### 2.2. Study Design

At enrollment, all subjects underwent anthropometric measurements and a cardiopulmonary exercise test, according to previously reported procedures [15]. Repeated measures of energy availability (EA) were obtained during 3 different periods in the season. The effects on EA of supplementary CHO intake during exercise or a nutritional intervention were assessed in Phase 2 and Phase 3, respectively.

#### 2.2.1. Phase 1

Phase 1 involved a baseline assessment (4-day basal assessment). In the “early pre-season” period (December), athletes underwent a moderate training load (~10 h/week, ~600 power stress score [PSS]/week). During this period, the athletes continued their usual dietary habits to provide a baseline assessment without the experimental interventions.

#### 2.2.2. Phase 2

Phase 2 involved supplementary CHO intake during exercise. This phase was performed during the “late pre-season” training period, which involved a heavy training load (~15 h/week, ~900–950 PSS/week), typically undertaken in January, before the start of the cycling races (end of February). Phase 2 was composed of 2 consecutive 4-day periods. During the first 4-day period, the dietary habits of the athletes were recorded (observation period); in the second 4-day period, carbohydrate supplementation during physical activity was introduced (supplementary CHO). More precisely, 30–60 g CHO/h for endurance exercise durations of 1–2.5 h and up to 90 g CHO/h for prolonged endurance exercise of >2.5–3 h were prescribed, as recommended by the American College of Sports Medicine (ACSM) (Table 1) [6]. Carbohydrate supplementations with a high glycemic index were administered in solid form and/or in liquid form (e.g., isotonic beverages containing maltodextrin).

#### 2.2.3. Phase 3

Phase 3 involved supplementary CHO intake during exercise with a daily nutritional intervention. This phase was performed during the competitive season (July). During this period, athletes underwent a light training load (~10 h/week, ~400–450 PSS/week), excluding competition days. Phase 3 was composed of an initial 4-day period of nutritional assessments, when the dietary habits of the athletes were simply recorded (observation period), and a second 4-day period involving a global nutritional intervention, when the daily prescribed energy intake (EI) was tailored to the needs of each individual athlete, according to the guidelines and related endurance training program [6]. Briefly, for moderate exercise programs (1 h/d), 5 to 7 g/kg/d of CHOs was prescribed; for endurance programs (1–3 h/d) involving moderate–high intensity exercise, 6–10 g/kg/d of CHOs was prescribed; for extreme commitment (>4–5 h/day) involving moderate–high intensity exercise, 8–12 g/kg/d of CHOs was recommended (Table 1). Dietary protein intake ranged from 1.2 to 2.0 g/kg/d. Fat intake ranged from 20% to 35% of the total energy intake [6]. In addition, 1 to 4 h before exercise, 1–4 g/kg of CHOs was recommended; after exercise, a CHO intake of 0.6–1.0 g CHO/kg/h during the first 30 min and again, every 2 h for 4–6 h, was then recommended, to adequately replace the athlete’s glycogen stores [6].

### 2.3. Measurement of Training and Exercise Energy Expenditure (EEE)

All the participants followed individualized training plans (for training duration and intensity), designated by their team’s coach. Mechanical work values were continuously recorded from the participant’s crank-based power meters [16], while the data on the environmental parameters (air temperature and global positioning system, GPS) were collected by smartwatches. All the data were automatically uploaded to a telemetry platform (SelfLoops SNC, Fermo, FM, Italy). The EEE during the cycling sessions was then calculated using the validated methodology [17,18]:

EEE (kcal) = Average Power (W) × Duration (Hours)/(4.186 × 0.22)

The training load was evaluated using the power stress score (PSS), which was calculated from the power data assessing the intensity and duration, in seconds, of each training session (t) and the individual functional threshold power (FTP), as follows:

PSS = (t × EP × IF)/(FTP × 3600) × 100

where FTP is the highest average power that can be sustained for approximately one hour [19]; EP is the effective power, a weighted average power that considers ride variability; IF is the intensity factor, calculated as the ratio between the EP of the athlete and his FTP. A PSS of 100 represents an athlete’s ride at his FTP for an hour.

### 2.4. Quantification of Dietary Intake (EI)

Food diaries and photographic records of each meal eaten by the athletes were used to calculate their caloric intake and to assess the amounts of macronutrients consumed across the 4-day periods. The diaries were delivered daily by the athlete to the coach, who verified whether the information was complete. At the end of the period, the diaries and photos were given to the dietitians. The athlete’s dietary intake was then analyzed individually by 2 dietitians, using dietary analysis software (Metadieta, v 4.7, METEDA S.r.l., Roma, Italy). The output values were then averaged to provide estimates of the EI, in kilocalories per day (kcal/day).

### 2.5. Calculation of Energy Availability (EA)

Energy availability (EA) was calculated as [20,21]:

EA = (EI − EEE)/Fat Free Mass (FFM)

The FFM and body composition were estimated using bioelectrical impedance analysis (BIA). EA values less than 30 kcal/kg FFM/day were considered as low energy availability (LEA) [9,21].

### 2.6. Continuous Glucose Monitoring

The athlete’s interstitial glucose (IG) profile was monitored using a minimally invasive method, namely a biosensor (CGM, Supersapiens, Freestyle Libre Sense^®^, Abbott, Chicago, IL, USA) [22], positioned on the back of the upper arm. The device allows the recording of data in the range between 55 and 200 mg/dL. The biosensor records the person’s glucose concentration every minute and the data were recorded on the athlete’s phone, which was connected via Bluetooth. Only data collected at a frequency of at least 4 samples per hour were assessed. When data gaps occurred during a day of measurements, the data from that day were excluded from the analysis. The average glucose concentration and glucose variability (standard deviation and coefficient of variation) were assessed: (i) during 24 h (overall average data, from midnight to midnight); (ii) during nighttime (from 23.00 to 7.00); and (iii) during exercise. The percentage of time spent below the glucose concentration range [TBR, <70 mg/dL or <3.9 mmol/L], within the glucose range [TIR, 70–140 mg/dL or 3.9–7.8 mmol/L], or above the range [TAR, >140 md/dL or >7.8 mmol/L]), were calculated and expressed as percentages. Episodes of hypoglycemia lasting at least 15 min during 24 h were also recorded. At least 2 sensor values >70 mg/dL that were ≥15 min apart, with no intervening values < 70 mg/dL, were necessary to end an event considered “below range” [23].

### 2.7. Statistical Analysis

The sample size was calculated to assess differences in the 24 h glucose level standard deviation (SD), a metric derived by the CGM, based on data obtained from a healthy population [24]. A sample of 8 athletes provided 80% of the power to reject the null hypothesis, assuming an average intra-individual change in the SD of 3% between the observation and intervention assessments, using a two-sided type I error of 0.05.

The data are expressed as the mean ± SD for continuous variables. Categorical variables are presented as counts and percentages. The normal distribution of the continuous variables was checked using skewness and kurtosis measures. Changes in continuous or dichotomic variables were quantified using a paired *t*-test or a chi-squared test, respectively. Determinants of LEA occurrence and 24 h glucose variability were investigated using logistic and linear regression analyses, with training load, energy intake, free-fat mass, and air temperature included in the models.

The statistical analyses were conducted using IBM SPSS Statistics (v. 28.0.0.0, IBM, Armonk, NY, USA). Significance was set at *p* < 0.05 for all the statistical tests.

## 3. Results

The characteristics of the participants at enrollment are reported in Table 2. One cyclist withdrew from Phase 3 of the study, due to an injury.

### 3.1. Baseline Assessment (Phase 1)

The data collected during the “early pre-season” period (Phase 1) are presented in Table 3. The analysis revealed that during this phase, three out of eight athletes were experiencing LEA.

**Figure 1:**
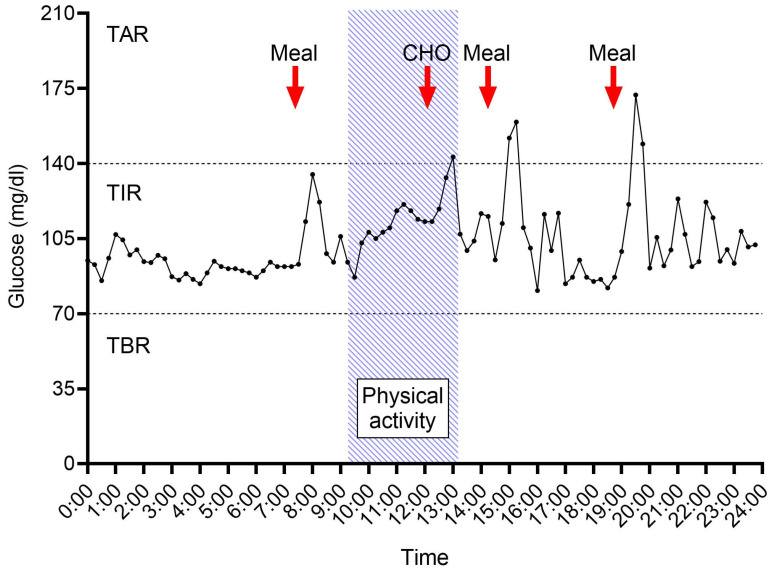
Glucose trend in an athlete during a training day (representative case). The period of physical activity (between 9.00 and 13.00) is marked in blue. The intake of the 3 main meals (Meal) and the moment of spontaneous supplementation with CHOs during exercise are also indicated with a red arrow. Abbreviations: TAR, time above the glucose level of 140 mg/dL; TIR, time in the range between 70 and 140 mg/dL; TBR, time below the glucose level of 70 mg/dL.

The glucose values recorded during 24 h are shown in Table 3. On average, two periods of hypoglycemia per day were recorded for each athlete during 24 h. In the whole cohort, the TBR averaged 5.4 ± 13.5% during the nighttime and 1.5 ± 2.9% during the daytime (Figure 1).

### 3.2. Supplementary CHO Intake (Phase 2)

**Figure 2:**
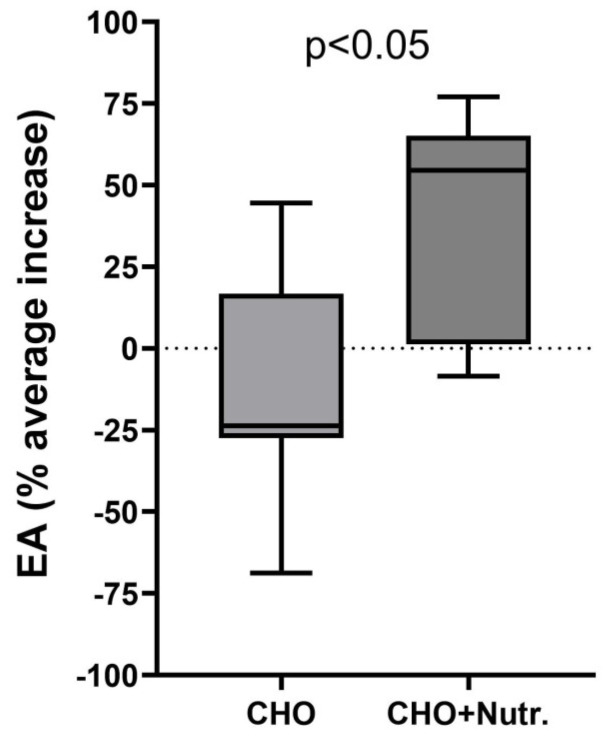
Effect of 2 different dietary regimes on the percentage increase in energy availability (EA) in athletes. The length of the box is the difference between the 75th and 25th percentiles; the horizontal line inside the box is the median value; the ends of the box are the 9–95th percentiles. CHO = carbohydrate supplementation during physical activity. CHO+Nutr = carbohydrate supplementation during physical activity + global nutritional intervention.

During the late pre-season period (Phase 2), the balance between EI and EEE showed that all eight of the investigated cyclists were experiencing LEA during the 4-day basal assessment. During supplementary CHO correction where the EA was not modified (Figure 2), seven out of the eight athletes continued to experience LEA.

The average glucose values measured during 24 h, during nighttime, and during exercise, were not altered by supplementary CHO correction (Table 3). Conversely, a significant increase in glycemic variability (both SD and CV) was observed during the intervention period. Episodes of hypoglycemia during 24 h and the TBR during both the daytime and the nighttime were not altered by the supplementary CHO correction. However, the TAR was increased during the daytime.

### 3.3. Supplementary CHO Intake with Daily Nutritional Intervention (Phase 3)

During the competitive season, the studies were carried out on days with a light training load, excluding competition days. During the baseline observations, five out of seven athletes were experiencing LEA. The global nutritional intervention, tailored to the needs of each individual athlete according to the relevant guidelines, resulted in marked changes to EA, because only one out of seven athletes was found to be experiencing LEA (Figure 2).

The average values in terms of the glucose level, episodes of hypoglycemia, and TBR were not altered by the global nutritional intervention (Table 3).

### 3.4. Determinants of LEA and Glucose Variability

When all the observations collected during the three phases of the study were considered as a whole group, multivariate logistic regression analysis of LEA was, as expected, found to be positively influenced by the training load (OR 1.06, 95% CI 1.03 to 1.09), EI (OR 0.994, 0.991 to 0.997), and FFM (OR 1.46, 1.04 to 2.04), independent from the air temperature (OR 1.06, 0.98 to 1.16).

**Figure 3:**
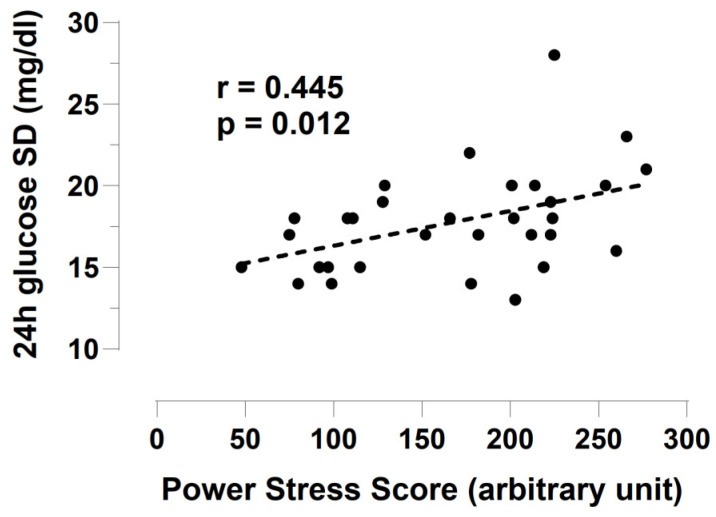
Relationship between the training load (expressed as the power stress score) and the variability of the interstitial glucose, expressed as the standard deviation, using linear regression analysis.

The multivariate linear regression analysis showed that the glucose variability (expressed by the 24 h standard deviation) was positively associated with the training load (B 0.014, 95% CI 0.004 to 0.025) and FFM (0.487, 0.215 to 0.759) and negatively associated with EI (−0.001, −0.002 to −0.001). The glucose variability was not affected by the air temperature. The results of the linear regression between the 24 h glucose SD and the training load are reported in Figure 3.

## 4. Discussion

The main results in the present study are that: (1) LEA is highly frequent in adolescent cyclists during all periods of the competitive season; (2) supplementary CHO intake during exercise, based on the intensity and duration of the exercise, does not modify LEA prevalence; and (3) only the global nutritional intervention, tailored to the needs of each individual athlete, according to the relevant guidelines, resulted in a marked reduction in LEA.

### 4.1. Energy Availability in Adolescent Athletes

During Phase 1, the adolescents continued their usual nutritional habits without any external interventions. During this preliminary phase, three out of eight athletes were already experiencing LEA, which clearly highlighted their lack of knowledge about how to balance their nutritional intake according to the duration and intensity of the exercise taking place. The problem related to their lack of knowledge emerges even more clearly over the course of the season, when the exercise intensity increases. In fact, during the observation period in Phase 2, when the workload increased, all the adolescent athletes were experiencing LEA. However, a lack of knowledge cannot be assumed to be the only reason. Many athletes and people in general have adequate nutritional knowledge, but choose not to apply it, or lack the personal agency/motivation to do so [25]. In particular, as previously stated, many cyclists fear that an increase in body weight may limit the weight/power ratio and often adopt dietary strategies aimed at limiting their body weight. Promoting adequate knowledge of nutrition among adolescents should be an important part of the school curriculum. A person’s knowledge of nutrition can in fact play a small, but fundamental, role in the adoption of healthier eating habits [26].

A recent review by Gould et al. [27] highlighted that LEA among adolescents is common, although the majority of the research was carried out with females. Studies carried out on adolescents (<18 years old) have reported a variable prevalence of LEA according to the sport discipline and sex, with a prevalence of 6–60% among females and 10–47% among males, suggesting that the risk of LEA in adolescents is comparable to, or even higher than, that in adults, but no information is given regarding cyclists (Table 4). Among male adolescent athletes, EA has been investigated in regard to climbing [28], cross-country running [29,30], soccer [31], and rink hockey [32]. Koehler et al. [33] found LEA in 56% of 167 male adolescent and young adult athletes involved in various sport disciplines. To our knowledge, the present study is the first to pay particular attention to LEA in male adolescent cyclists. Energy expenditure in cycling is highly variable throughout the season, which means that spot observations are useless. In fact, Viner et al. [34] observed that EA in adult road cyclists may vary from 21.7 kcal/kg FFM/day during the off-season to 18.8 kcal/kg FFM/day during the pre-season and 19.5 kcal/kg FFM/day during the competition season. Also, in our cohort, differences in EA were conditional upon the different workloads during the observation phases.

Some methodological aspects make the calculation of EA critical. Competitive athletes may experience high heterogeneity in terms of their daily EA, even during a limited observation period [35,36,37,38], and a detection period of at least 3 days is necessary to limit variability within these days. As regards assessments of the variables, such as the energy expenditure and free-fat mass, they should be measured directly, rather than using indirect estimations [39]. Surrogate measures of LEA were adopted to estimate the effects of prolonged exposure to LEA, such as the risk of injuries or time lost due to injuries [40,41,42]. In our study, the athlete who was unable to participate in Phase 3 of the study due to injury, was experiencing LEA during both phases 1 and 2.

Finally, studies conducted on adult endurance athletes, during different phases of the season, have shown that LEA is mainly due to low EI, and especially to low CHO intake, because of a failure to comply with the relevant carbohydrate intake recommendations or due to voluntary restrictions related to nutritional intake [34]. Accordingly, the impact of two different nutritional strategies (CHO supplementation during physical activity or a nutritional approach) clearly emerges in the present study. With the increase in CHO supplementation during physical activity, seven athletes continued to experience LEA. Conversely, when a daily nutritional approach tailored to the needs of each individual athlete was also implemented, during the competitive season, only one athlete continued to experience LEA. A summary of the previous studies in this area is presented in Table 4.

**Table 4:** Studies assessing low energy availability (<30 kcal/kg FFM/day) by measuring energy intake (EI), exercise energy expenditure (EEE), and free-fat mass (FFM) in adolescent athletes.

Author	Year	*n*	Age (Range)	Sport	EI	EEE (Estimated/Measured)	FFM (Assessment Method)	Detection Length	LEA Prevalence
Men									
Silva et al. [32]	2016	34	12–16	Rink hockey	Diet logs	Estimated [43]	Skinfold method	3 d	10%
Cherian et al. [31]	2018	11	10–18	Junior soccer	Direct calorimetry	Measured	Skinfold method	4 d	24%
Matt et al. [29]	2021	12	14–17	Cross-country running	Block FFQ	Measured	DEXA	4 w	30%
Simič et al. [28]	2022	13	13–18	Climbing	Diet logs	Estimated [43]	Bio-impedance analysis	3 d	47%
Women									
Hoch et al. [44]	2009	80	13–18	Various disciplines	Diet logs	Estimated [45]	DEXA	3 d	6%
Silva et al. [46]	2015	36	16–18	Rhythmic gymnastics	24 h recall diet log	Estimated [43]	Bio-impedance analysis	1–4 d	45%
Braun et al. [47]	2017	56	13–17	Football	Diet logs	Estimated [43]	Doubly labelled water	7 d	53%
Cherian et al. [31]	2018	10	10–18	Junior football	Direct calorimetry	Measured	Skinfold method	4 d	58%
Civil et al. [48]	2018	20	17–19	Ballet dance	Diet logs and 24 h recall	Measured	DEXA	7 d	22%
Matt et al. [29]	2021	60	14–17	Cross-country running	Block FFQ	Measured	DEXA	4 w	60%
Simič et al. [28]	2022	14	13–18	Climbing	Diet logs	Estimated [43]	Bio-impedance analysis	3 d	53%

Abbreviations: FFQ, food frequency questionnaire; DEXA, dual-energy X-ray absorptiometry.

In addition, low spontaneous carbohydrate intake is accompanied by an adequate or high intake of protein, sometimes considered by cyclists as a macronutrient that does not cause weight gain [21]. Although some studies have evaluated the favorable impact of educational sessions on energy availability [49,50], the present study is the first to document these effects in a population of elite adolescent athletes.

### 4.2. LEA, Glucose Dynamics, and the Environment

Generally, carbohydrate metabolism during endurance exercise has a critical role in energy production. Three days of endurance training accompanied by low EA was found to deplete the muscle glycogen content by about 30% [51]. Furthermore, the blood glucose concentration in blood samples collected after exercise was significantly reduced in athletes with LEA [52]. No differences were observed in LEA athletes at rest [52,53]. However, the influence of low EA on exogenous carbohydrate oxidation during endurance exercise remains unclear.

Two studies have evaluated the impact of a state of LEA on interstitial glucose with CGMs and were conducted on healthy military personnel [54] and elite race walkers [55]. Acute exposure to LEA was found to increase the occurrence of hypoglycemic episodes. More specifically, in 23 healthy military personnel, who increased their usual daily EEE for 2 days, the percentage of time spent in mild, not severe, hypoglycemia was higher when they consumed a diet designed to induce 93% hypoglycemia, than when their caloric intake was designed to maintain an energy balance [54]. In a group of 10 elite race walkers, the mean nocturnal interstitial glucose concentration, total interstitial glucose area under the curve, and nocturnal interstitial glucose variability, were not different between the 9-day period spent in LEA and the control dietary intervention [55].

In our cohort, the mean nocturnal and 24 h interstitial glucose concentrations were also not affected by LEA, as previously observed [55]. Conversely, different from what was observed during an acutely induced LEA condition [54], the time spent in mild hypoglycemia (TBR, below the threshold of 70 mg/dl of glucose) was found to not be affected by LEA. Finally, the nocturnal interstitial glucose variability was observed to be reduced when athletes were experiencing LEA. However, the present observations were carried out in the field, during different phases of the competitive season, and were characterized by different energy intake, workloads, and environmental conditions (temperature, humidity); in addition, these results cannot be generalized due to the low number of observations.

Environmental conditions are extremely variable during the competitive season and may affect the blood glucose of athletes. A metanalysis including over 230,000 participants in 15 countries, reported peak of values in winter and a nadir in summer [56]. In our study, which adopted continuous 24 h measurement, no relationships were observed between air temperature during exercise and the 24 h average glucose level or 24 h glucose variability.

### 4.3. Clinical Implications

In the present study, the repetitive observation of LEA in adolescent athletes during different periods of the competitive season clearly underscores a lack of nutritional education among adolescents. To optimize performance and achieve a greater power-to-weight ratio, athletes pay more attention to their body weight, which is easy to manipulate [42]. This perception is also strongly rooted in the approach taken by coaches. The occurrence of cyclists showcasing increased performance with increased body weight (increased muscle mass due to appropriately increased food intake) is still cited as an anecdotal event.

In addition, an estimated prevalence of 9% of adolescent endurance athletes were found to have eating disorders [57,58,59]. At this age, athletes become more autonomous and their primary attention to their body weight, added to a low level of nutritional education, could pave the way to the adoption of incorrect eating habits in future. This is why greater attention should be paid to educating adolescent athletes rather than adopting short-term performance optimization strategies. Recent literature has described the possibility of increasing the performance of athletes by inducing a short-term state of LEA, called adaptive LEA [21]. However, repeated findings in our cohort, especially outside the competition season, highlight bad practices in terms of weight management rather than a choice aimed at maximizing short-term performance.

### 4.4. Limitations

The first limitation is the small sample size. In particular, eight participants were necessary and only seven were assessed in Phase 3. Despite this, the difference between the two treatment regimens in Phase 3, in terms of LEA prevalence, was evident. In addition, this sample is highly homogeneous because the athletes belong to the same competitive team, have comparable training load, and have similar nutritional habits during the season. These characteristics ensured that the nutritional intervention was effective.

Second, reporting bias could impact the accuracy of the results. Regarding the assessment of energy expenditure, reporting bias was limited due to the use of an activity tracker. Likewise, reporting bias in regard to clinical nutrition was limited by the photographic record taken of each meal.

## 5. Conclusions

In conclusion, LEA is frequently found in adolescent cyclists during the different phases of the competitive season; the present study highlights the need for a global approach to nutrition management. The support of a nutrition professional in cycling teams is, therefore, important for education and nutrition management involving endurance athletes.

## Figures and Tables

**Table 1:** Prescribed carbohydrate (CHO) intake during experimental interventions in Phase 2 and Phase 3.

	Phase 2 Supplementary CHO (Only During Exercise)	Phase 3 Nutritional Intervention (Daily)
***Pre-exercise (1–4 h)***		
	free	1–4 g/kg
***During exercise***		
duration 1–2.5 h	30–60 g	30–60 g
duration >2.5–3 h	up to 90 g	up to 90 g
***After exercise (up to 6 h)***		
	free	0.6–1.0 g/kg/h
***Daily CHO intake***		
1–3 h/d training	free	6–10 g/kg/d
4–5 h/d training	free	8–12 g/kg/d

**Table 2:** Baseline characteristics of the participants.

Parameters	Mean ± SD (Range)
Age (years)	16.6 ± 0.5 (16–17)
Weight (kg)	62.5 ± 3.4 (55.5–67)
Height (cm)	175.8 ± 4.1 (171–183)
BMI (kg/m^2^)	20.1 ± 0.9 (18.5–21.5)
SBP (mmHg)	116.9 ± 5.9 (110–125)
DBP (mmHg)	60.0 ± 5.3 (50–70)
VO_2_max (mL/min/kg)	67.0 ± 4.5 (58.1–72.1)

Abbreviations: BMI, body mass index; SBP, systolic blood pressure; DBP, diastolic blood pressure; VO_2_max, maximum oxygen uptake.

**Table 3:** Training metrics, energy assessment, and glucose parameters during the 3 study phases (data are expressed as mean ± SD).

	Phase 1	Phase 2			Phase 3		
	*Observation*	*Observation*	*Intervention*	*p =*	*Observation*	*Intervention*	*p* =
	*n* = 8	*n* = 8	*n* = 8		*n* = 7	*n* = 7	
PSS (AU)	191 ± 39	211 ± 35	243 ± 34	0.109	90 ± 25	91 ± 34	0.968
Intensity factor (%)	79 ± 6	76 ± 4	82 ± 6	0.051	65 ± 5	61 ± 5	0.354
CHO intake (g/kg/d)	7.1 ± 2.1	5.4 ± 0.8	7.2 ± 1.5	0.052	6.0 ± 0.7	8.1 ± 0.8	0.001
Protein intake (g/kg/d)	2.1 ± 0.8	1.8 ± 0.7	1.8 ± 0.3	0.888	1.6 ± 0.2	1.7 ± 0.3	0.076
Fat intake (% of EI)	31.0 ± 6.7	28.4 ± 6.4	27.6 ± 4.1	0.980	23.3 ± 7.3	23.3 ± 7.1	0.986
EEE (kcal/day)	1867 ± 242	2304 ± 384	2187 ± 353	0.146	1121 ± 388	1268 ± 239	0.419
EI (kcal/day)	3403 ± 829	2749 ± 328	3204 ± 489	0.028	2678 ± 346	3284 ± 349	0.006
FFM (kg)	54 ± 3	56 ± 3	56 ± 3	0.685	53 ± 2	53 ± 2	0.356
EA (kcal/kg FFM/day)	33 ± 15	21 ± 5	18 ± 8	0.343	29 ± 9	38 ± 6	0.040
Subjects experiencing LEA	3	8	7	0.350	5	1	0.001
**Glucose Parameters**							
Mean 24 h (mg/dL)	109 ± 9	105 ± 5	111 ± 13	0.319	105 ± 7	104 ± 10	0.619
Mean exercise (mg/dL)	123 ± 18	120 ± 8	133 ± 21	0.328	109 ± 9	106 ± 13	0.518
Mean nighttime (mg/dL)	97 ± 9	96 ± 8	96 ± 8	0.854	96 ± 6	95 ± 9	0.653
SD 24 h (mg/dL)	18 ± 3	17 ± 2	21 ± 4	0.024	15 ± 2	17 ± 2	0.249
SD exercise (mg/dL)	15 ± 5	16 ± 4	17 ± 3	0.579	12 ± 2	14 ± 3	0.137
SD nighttime (mg/dL)	11 ± 4	9 ± 3	7 ± 2	0.376	8 ± 2	8 ± 2	0.768
TAR daytime (%)	11.8 ± 11.6	6.5 ± 5.1	25.9 ± 23.7	<0.001	6.6 ± 3.8	6.6 ± 4.6	0.497
TAR nighttime (%)	1.0 ± 3.0	0.5 ± 1.0	0.6 ± 1.8	0.775	0.2 ± 0.6	0.8 ± 2.0	0.116
TIR daytime (%)	86.6 ± 11.5	92.5 ± 5.4	72.4 ± 22.6	<0.001	91.3 ± 6.4	90.1 ± 5.9	0.497
TIR nighttime (%)	93.4 ± 14.2	97.0 ± 4.7	95.7 ± 6.7	0.449	98.9 ± 2.2	95.3 ± 10.3	0.106
TBR daytime (%)	1.5 ± 2.9	0.9 ± 1.7	1.4 ± 2.8	0.498	2.0 ± 6.1	3.1 ± 5.4	0.499
TBR nighttime (%)	5.4 ± 13.5	2.5 ± 4.0	3.5 ± 6.3	0.498	0.9 ± 2.1	3.6 ± 9.6	0.192
Hypo/subject/day	2.1 ± 5.0	0.5 ± 1.0	1.1 ± 2.4	0.233	0.5 ± 0.9	2.7 ± 5.7	0.057

Abbreviations: PSS, power stress score; AU, arbitrary unit; CHO, carbohydrate; EEE, exercise energy expenditure; EI, energy intake; FFM, free-fat mass; EA, energy availability; LEA, low energy availability (<30 kcal/kg FFM/day); SD, standard deviation; TAR, time above range; TIR, time in range; TBR, time below range; Hypo, episodes of hypoglycemia (glucose <70 mg/dL for at least 15 min). *p* relates to a two-tailed paired *t*-test for continuous variables and a chi-squared test for categorical variables.

## Data Availability

The data are available upon reasonable request.
